# Adrenergic inhibition facilitates normalization of extracellular potassium after cortical spreading depolarization

**DOI:** 10.1038/s41598-021-87609-w

**Published:** 2021-04-14

**Authors:** Hiromu Monai, Shinnosuke Koketsu, Yoshiaki Shinohara, Takatoshi Ueki, Peter Kusk, Natalie L. Hauglund, Andrew J. Samson, Maiken Nedergaard, Hajime Hirase

**Affiliations:** 1 Laboratory for Neuron-Glia Circuitry, RIKEN Center for Brain Science, Wako, Saitama 351-0198 Japan; 2 Faculty of Core Research Natural Science Division, Ochanomizu University, Bunkyo-Ku, Tokyo, 112-8610 Japan; 3 Department of Integrative Anatomy, Nagoya City University Graduate School of Medical Sciences, Nagoya, Aichi 467-8601 Japan; 4 Division of Histology and Cell Biology, Department of Anatomy, Jichi Medical University, Shimotsuke, Tochigi 329-0498 Japan; 5 Center for Translational Neuromedicine, Faculty of Health and Medical Sciences, University of Copenhagen, 2200 Copenhagen, Denmark; 6 Center for Translational Neuromedicine, University of Rochester Medical Center, Elmwood Avenue 601, Rochester, NY 14642 USA

**Keywords:** Migraine, Stroke, Astrocyte, Neuroscience, Diseases of the nervous system

## Abstract

Cortical spreading depolarization (CSD) is a propagating wave of tissue depolarization characterized by a large increase of extracellular potassium concentration and prolonged subsequent electrical silencing of neurons. Waves of CSD arise spontaneously in various acute neurological settings, including migraine aura and ischemic stroke. Recently, we have reported that pan-inhibition of adrenergic receptors (AdRs) facilitates the normalization of extracellular potassium after acute photothrombotic stroke in mice. Here, we have extended that mechanistic study to ask whether AdR antagonists also modify the dynamics of KCl-induced CSD and post-CSD recovery in vivo. Spontaneous neural activity and KCl-induced CSD were visualized by cortex-wide transcranial Ca^2+^ imaging in G-CaMP7 transgenic mice. AdR antagonism decreased the recurrence of CSD waves and accelerated the post-CSD recovery of neural activity. Two-photon imaging revealed that astrocytes exhibited aberrant Ca^2+^ signaling after passage of the CSD wave. This astrocytic Ca^2+^ activity was diminished by the AdR antagonists. Furthermore, AdR pan-antagonism facilitated the normalization of the extracellular potassium level after CSD, which paralleled the recovery of neural activity. These observations add support to the proposal that neuroprotective effects of AdR pan-antagonism arise from accelerated normalization of extracellular K^+^ levels in the setting of acute brain injury.

## Introduction

Cortical spreading depolarization (CSD) is a self-propagating wave of depolarization that travels across the cerebral cortex with a velocity of approximately 4 mm/min^1^. CSD is triggered by a local increase of the extracellular potassium level ([K^+^]_e_). While action potential-induced synaptic transmission is not a critical factor in the slow propagation of CSD waves, the elevated [K^+^]_e_ is considered to play a pivotal role in this phenomenon. A sufficient rise of [K^+^]_e_ depolarizes neurons and results in enhanced release of neurotransmitters, including glutamate. The resultant depolarization leads to the opening of voltage-dependent K^+^ channels and activation of NMDA receptors, which together promote further K^+^ efflux to the extracellular space, thus contributing to the regenerative elevation of [K^+^]_e_^2–6^. Along with altered concentrations of other ions including Na^2+^ and Cl^-^, CSD provokes a near-complete breakdown of transmembrane ion gradients^7^. After the passage of a CSD wave, the high prevailing [K^+^]_e_ induces long-lasting membrane depolarization that inactivates voltage-gated sodium channels, resulting in prolonged electrical silencing of neurons, as supported by computer simulation of ion conductances in model neurons^8^. Accordingly, the normalization of [K^+^]_e_ is considered a precondition for the de-inactivation of the sodium channels and resultant the recovery of neuronal responsiveness. Additionally, other factors contribute to the depression of activity after CSD that typically outlasts the neuronal depolarization, such as intracellular Zn^2+^ and Ca^2+^ accumulation, extracellular adenosine accumulation, and/or Na^+^/K^+^-ATPase activation^9,10^.

Spreading depolarization occurs in a diverse spectrum from transient spreading depolarizations that occur in metabolically intact tissue (e.g., migraine aura) to terminal spreading depolarization in severely ischemic tissue (e.g., ischemic stroke, subarachnoid hemorrhage, and traumatic brain injury, TBI)^7,11,12^. Multiple studies have shown that the turnover of interstitial (extracellular) fluid in the cortical parenchyma is compromised in animal models of TBI and ischemia^13–15^. In the case of stroke, CSD induces cytotoxic edema of neurons due to the imbalance of ionic concentrations^16^. Stagnant interstitial fluid contains high K^+^, which induces recurring CSDs and contributes to edema formation in ischemia^17^ that can lead to secondary brain injury. We have recently reported that pan-adrenergic receptor (AdR) antagonism by a cocktail of prazosin, atipamezole, and propranolol reduces the extent of infarction in mice after photothrombotic stroke by enhancing the normalization of [K^+^]_e_^18^. AdR blockers have previously been investigated as a migraine prophylaxis based on their effects on the generation and propagation of KCl-induced CSD in rats. Accordingly, acute topical application of the beta AdR antagonist propranolol decreased the occurrence of CSD^19^ and chronic administration of propranolol decreased the frequency of CSD episodes and slowed their propagation speed across cortex^20^. However, the mechanism for post-CSD recovery of neural activity has not hitherto been addressed in detail. Here, we investigated the effect of pan-AdR antagonism on the recovery of post-CSD spontaneous and evoked neural activities using the KCl-induced CSD paradigm.

Astrocytes represent a significant cellular component of the neuropil^21–24^ and are critical for maintaining K^+^ homeostasis^25^. In this study, we evaluated post-CSD induced neural activities by imaging in brain of living transgenic mice expressing the G-CaMP7 Ca^2+^ sensor in neurons and astrocytes (BAC-GLT-1-G-CaMP7 #817, G7NG817)^26^. We demonstrate that AdR antagonism facilitates the recovery of neural activity after KCl-induced CSD. Recordings of [K^+^]_e_ and sensory evoked neural activity in the somatosensory cortex showed that normalization of [K^+^]_e_ and recovery of neural activity occurred in parallel, and were accelerated in concert by AdR pan-antagonism. These observations highlight the therapeutic potential of AdR antagonism as a promoter of interstitial fluid ionic normalization in neurological conditions associated with recurring CSD.

## Results

### Transcranial imaging of G-CaMP mice reveals differential CSD propensities in AdR blocker-pretreated mice

**Figure 1:**
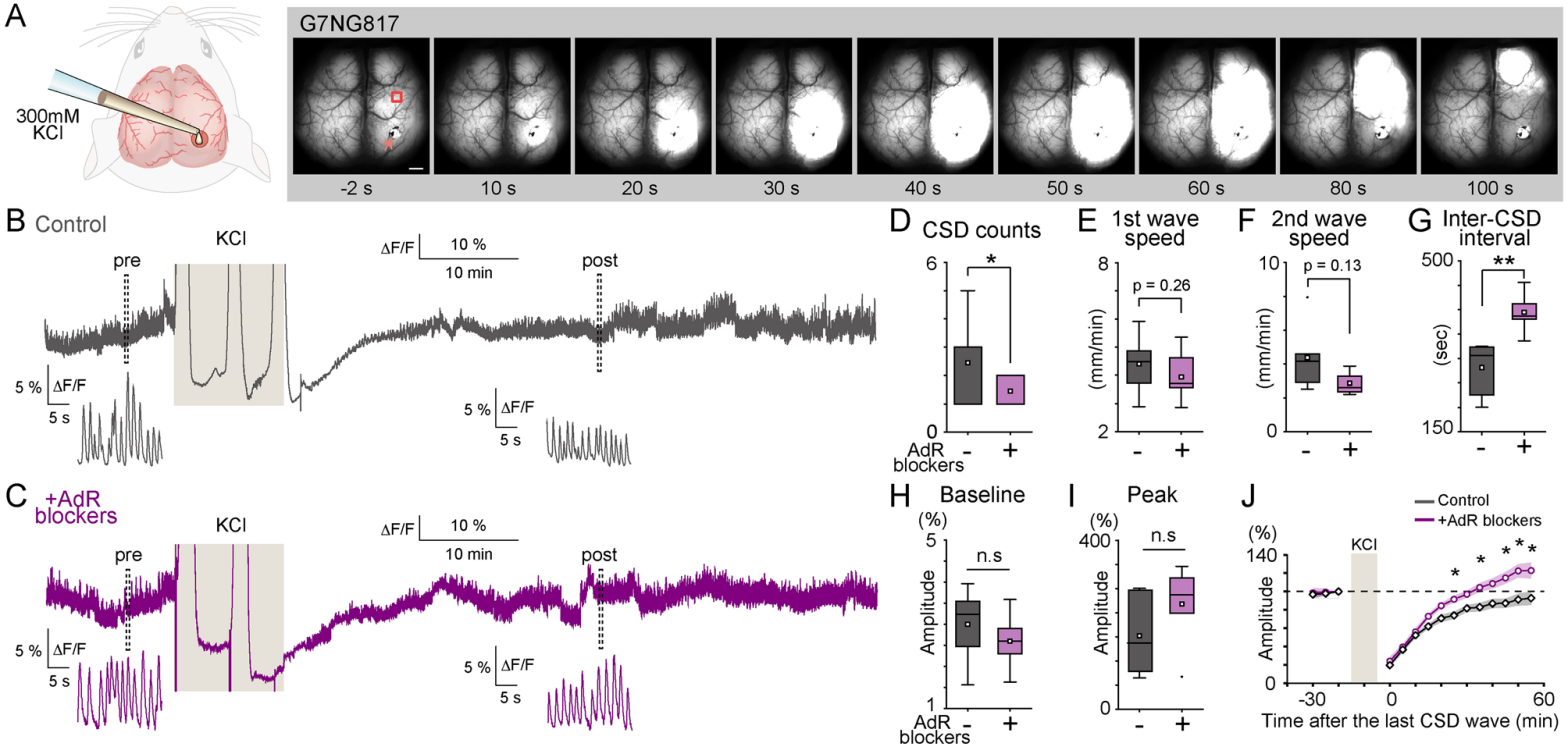
AdR antagonism suppresses CSD initiation and propagation. (**A**) Typical example of a CSD-associated Ca^2+^ wave in an anesthetized G7NG817 mouse observed by transcranial cortex-wide macro imaging. KCl (300 mM) was topically applied to a small craniotomy above the visual cortex at time 0. The yellow arrowhead points to the KCl application site. Representative Ca^2+^ dynamics shown in B and C were recorded in an ROI ~ 2 mm anterior to the KCl application site (red square). Scale bar 1 mm. (**B**) Example trace of Ca^2+^ signal (G-CaMP7 ΔF/F) from the same animal shown in (**A**). Insets are magnified plots of the respective pre- and post-periods marked by dotted rectangles showing UP/DOWN state slow oscillations that occur during urethane anesthesia. (**C**) Similar Ca^2+^ signal trace as (**A**). measured in a mouse pretreated with AdR pan-blockers. (**D**) Total count of CSD events. Untreated control: N = 9 mice, and AdR pretreated: N = 11 mice. (**E**) Propagation speed of the first CSD Ca^2+^ wave. Untreated vs. treated, 4.4 ± 0.4 *vs.* 3.9 ± 0.2 mm/min. (**F**) Propagation speed of the second CSD Ca^2+^ wave in mice multiple CSD waves were observed. Untreated control: N = 9 out of 11 mice, and AdR pretreated: N = 5 out of 11 mice. 4.4 ± 0.8 *vs.* 2.9 ± 0.3 mm/min. (**G**) Inter-CSD Ca^2+^ wave interval. (**H**) Comparison of baseline neural activity amplitude. ΔF/F: 3.0 ± 0.4% *vs.* 2.6 ± 0.3%, n.s. (**I**) Comparison of the peak CSD amplitude. ΔF/F: 202.4 ± 32.8% *vs.* 268.3 ± 33.9%, n.s. (**J**) Effect of preemptive AdR blocker administration on the recovery of spontaneous neural activity. Spontaneous activity amplitude recovers in 30–40 min after KCl removal in mice pretreated with AdR blockers (N = 8 mice), whereas recovery in untreated control mice takes 60 min. Relative amplitude of spontaneous cortical Ca^2+^ fluctuations before and after the last CSD wave passage (N = 8, normalized to the response 10 min before KCl). *p < 0.05, **p < 0.01, ***p < 0.001, error bars are SEM.

Macroscopic fluorescence imaging through the exposed skull allowed us to monitor spontaneous neural activities of the dorsal surface of the cortex using the G7NG817 mouse. Topical application of 300 mM KCl via a small craniotomy over the visual cortex induced a CSD wave that was detected as a sharp increase in Ca^2+^ signal. The CSD wave initiated within a minute of KCl application and propagated across the entire ipsilateral cortex (Fig. 1A, Supplementary Video S1), but was confined to the ipsilateral hemisphere, consistent with a literature report^27^. During the 10 min of KCl exposure, 6/9 mice had multiple CSD events, for a mean (SD) of 2.4 ± 0.4 CSD events. There was an apparent decrease of G-CaMP7 baseline signal decrement after CSD events possibly reflecting the pH-decrease (acidification) associated with the passage of CSD wave^28,29^. Pretreatment with AdR blockers significantly decreased the frequency of CSD occurrence (Fig. 1B–D) such that only 5/11 mice had multiple CSD events, for a mean of 1.5 ± 0.2 events in ten min (p = 0.036). AdR pan-antagonism did not result in obvious changes in CSD propagation speed of the first or second waves (Fig. 1E,F). However, AdR antagonism significantly prolonged the inter-CSD interval (Fig. 1G, 281.1 ± 22.0 s *vs.* 394.7 ± 19.7 s, p = 0.0044). The suppressive effect on CSD wave occurrences by AdR antagonism is likely to alter the threshold for CSD induction, despite a lack of effect on the pre-CSD slow wave activity (Fig. 1H). Additionally, we could not observe a significant CSD peak amplitude change by AdR antagonism (Fig. 1I); however, this is possibly due to the near-saturated sensitivity of G-CaMP7 at Ca^2+^ concentrations above 1 µM^30^.

Based on these observations of suppressive effects on CSD occurrence, we next asked whether AdR blockers also modulate post-CSD recovery of neural activity. We investigated the amplitude of the slow wave activity clearly recorded as 0.5–2 Hz fluctuations in the Ca^2+^ signal (UP/DOWN states) which essentially mirror local field potential (LFP) activity (Suppl. Fig. S1). These rhythmic Ca^2+^ activities were largely eliminated after the transit of CSD waves (Fig. 1B). After replacing KCl with artificial cerebrospinal fluid (ACSF), the magnitude of spontaneous activity gradually recovered to control levels within 60 min (N = 8 mice, Fig. 1B,J). Notably, administration of AdR blockers significantly enhanced the recovery of spontaneous activity after CSD (Fig. 1C, J, 90.7 ± 3.7% *vs.* 111.1 ± 7.5%, N = 8 mice for each group, p = 0.029 at 45 min, two-way repeated-measures ANOVA, F(1,14) = 4.11).

The enhanced post-CSD neural activity recovery in the AdR blocker-pretreated group could be explained by the decrease of CSD events in this group (Fig. 1D) resulting in less alterations of intracellular and extracellular environments. To address this, we compared post-CSD neural activity recovery in the non-treated control group that had one or two CSDs with the pretreated group that had two CSD events. Despite having equial or more CSD events, the enhanced recovery effect was present in the pretreated group (Supplementary Figure S2). Further, we find that recovery of spontaneous activity was not correlated to CSD event occurrence (30 min after KCl removal, Spearman’s rank correlation, control: r = − 0.02, p = 0.96; AdR treated: r = 0.26, p = 0.62). These analyses suggest that AdR blocker treatment has a prevailing effect on post-CSD neural activity recovery regardless of the number of CSD events induced in our experimental paradigm.

### Astrocytic Ca^2+^ activity is elevated after passage of CSD

**Figure 2:**
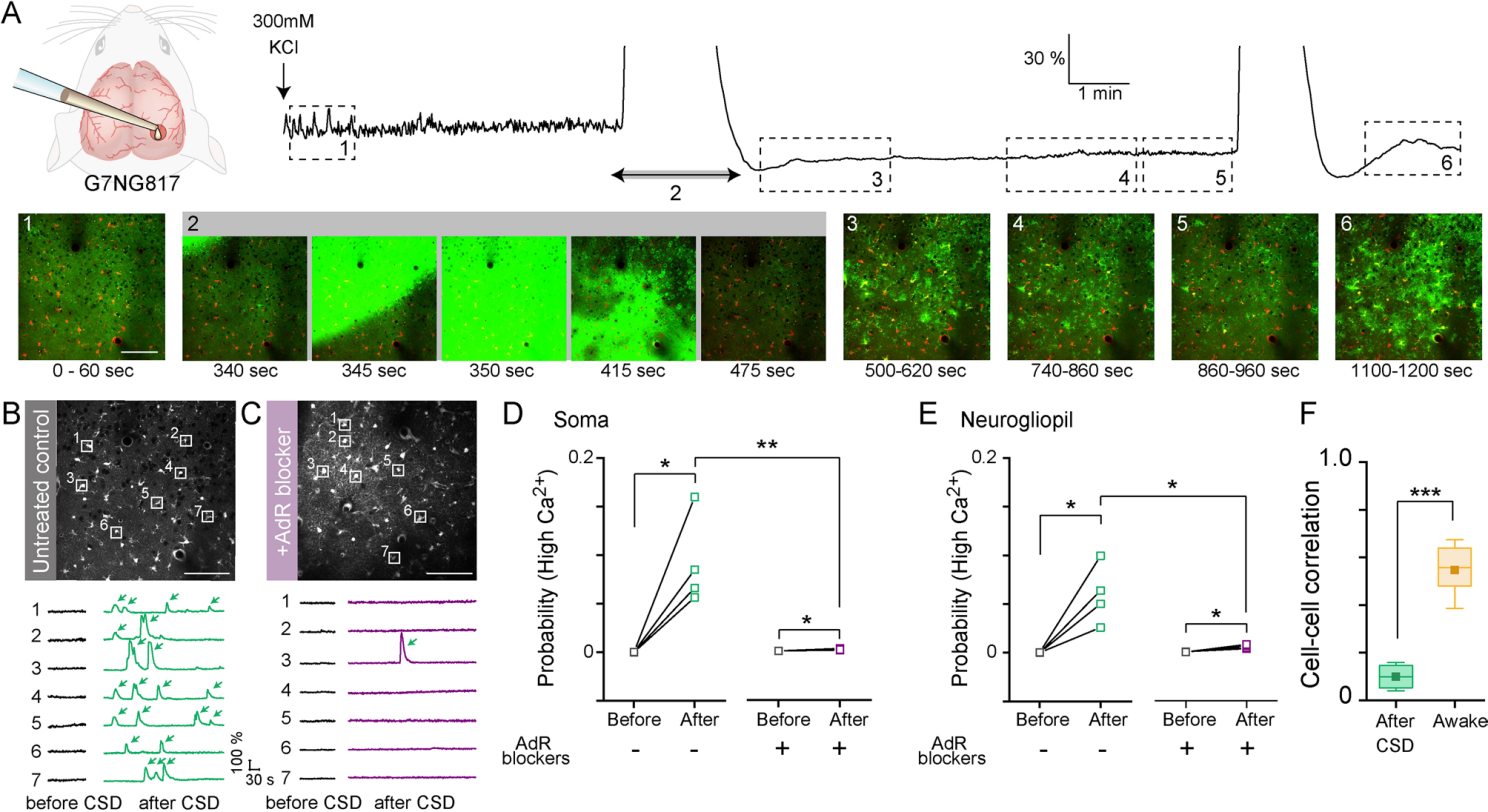
CSD-induced aberrant astrocytic Ca^2+^ activity is suppressed by AdR antagonism. (**A**) Two-photon imaging was performed to observe cellular and neuropil Ca^2+^ activity throughout the KCl-induced CSD experiment. (Top) The number on the dotted rectangles or the arrow indicated the time points when the images shown in the bottom panels were recorded. (Bottom) The images taken at 1, 3–6 are the maximum projections during the period and the time-series images taken at 2 are the corresponding raw images at each time. Scale bar 100 μm. (**B**) An example showing aberrant astrocytic Ca^2+^ activity (arrows) after the passage of the CSD Ca^2+^ wave in untreated naïve mice. (Left) Yellow squares on the astrocytic soma indicate the ROIs. (Right) Ca^2+^ traces of each ROI (ΔF/F). Black traces are in the pre-CSD period and the red traces are post-CSD periods. Scale bar: 100 μm. (**C**) An example showing that post-CSD-wave aberrant astrocytic Ca^2+^ activity is diminished in mice pretreated with AdR blockers (purple line). Scale bar 100 μm. (**D**,**E**) Quantification of post-CSD-wave aberrant astrocytic Ca^2+^ activity in soma (**D**) and neurogliopil (**E**) in G7NG817 mice (N = 4 mice) and G7NG817 mice pretreated with AdR blocker (N = 4 mice). (**F**) Comparison of the mean pairwise correlation coefficients for astrocytic Ca^2+^ activity per mouse (post-CSD: 198 cells from 4 mice; awake: 509 cells from 9 mice). *p < 0.05, **p < 0.01, ***p < 0.001.

To gain insight into the changes in cortical cellular activity during the recovery period after CSD, we next used two-photon microscopy to monitor the cellular Ca^2+^ dynamics in layer 2/3 of the somatosensory cortex. In the anesthetized control condition, neuropil signals fluctuated at a slow wave oscillation frequency similar to the macroscopic observations described above (Supplementary Video S2 left panel). CSD was observed as a large-amplitude Ca^2+^ signal wavefront that propagated in the neuropil (Fig. 2A, Supplementary Video S2 middle panel). Interestingly, astrocytic Ca^2+^ activities 15–300 s after the passage of the first CSD wave sharply increased, both in somata and processes (Fig. 2B,D,E. Soma: 1.03E−4 ± 1.68E−5 *vs.* 0.092 ± 0.023, p = 0.029; neurogliopil: 1.21E−4 ± 2.71E−5 *vs*. 0.060 ± 0.015, p = 0.030; Supplementary Video S2 right panel), consistent with a very recent description by Sugimoto et al.^31^ using a similar KCl CSD model. Remarkably, these aberrant post-CSD astrocytic somatic and neurogliopil Ca^2+^ activities were largely eliminated by pretreatment with AdR blockers (Fig. 2C–E, 0.0015 ± 3.90E−4, p = 0.0080). Neurogliopil activity was also substantially reduced by this treatment (Fig. 2E, 3.49E−4 ± 6.28E−5 *vs.* 0.056 ± 9.41E−4, p = 0.012).

To understand better the temporal coordination of the aberrant astrocytic Ca^2+^ activity, we calculated the pairwise correlation of simultaneously recorded Ca^2+^ signals of two different astrocytic somata. In line with an earlier observation using a Ca^2+^ indicator dye^32^, pairwise correlation coefficients were generally high during wakefulness. By contrast, a large majority of astrocyte pairs exhibited lower correlation values in the anesthetized condition post-CSD (Fig. 2F, 0.10 ± 0.028 *vs.* 0.55 ± 0.034, p = 5.8E−6). This result indicates that aberrant post-CSD astrocytic activity shares a similar desynchronization feature with that observed during ammonia intoxication^33^. Moreover, while post-CSD astrocytic activity was rescued by AdR pretreatment, its dynamics are distinct from the predominantly synchronous behavior in the awake condition.

### IP_3_R2KO delayed recovery from CSD and AdR antagonism facilitates CSD recovery in IP_3_R2 KO

**Figure 3:**
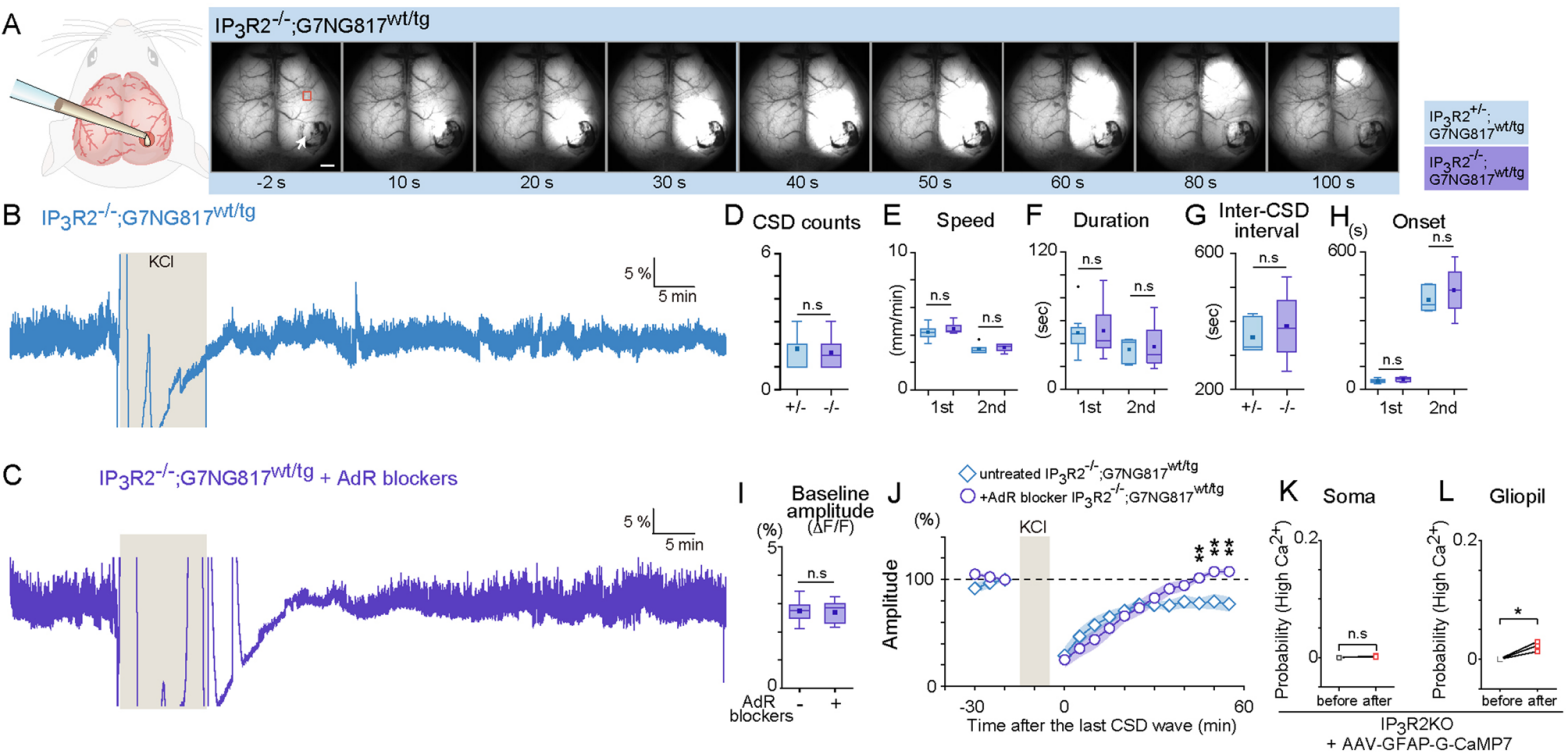
CSD propagation and neural activity recovery in IP_3_R2 KO mice. (**A**) Representative image of the time series of CSD propagation. Other than using IP_3_R2^−/−^;G7NG817^wt/tg^ double transgenic mouse as subjects, the experimental conditions are the same as in Fig. 1. Scale bar 1 mm. (**B**) Example trace of Ca^2+^ activity of an ROI located ~ 2 mm anterior to the KCl application site (Black square indicated in **A**). Note that neural activity does not recover completely within 50 min. (**C**) Similar Ca^2+^ signal trace as (**B**). measured in an IP_3_R2^−/−^;G7NG817^wt/tg^ mouse pretreated with AdR blockers. (**D**) Comparison of CSD Ca^2+^ wave number during 10 min KCl application between IP_3_R2^+/−^;G7NG817^wt/tg^ and IP_3_R2^−/−^:G7NG817^wt/tg^ mice. 1.8 ± 0.2 *vs.* 1.6 ± 0.3, from N = 10 *vs.* N = 8, p = 0.64. (**E**) Comparison of CSD Ca^2+^ propagation speed between IP_3_R2^+/−^;G7NG817^wt/tg^ and IP_3_R2^−/−^:G7NG817^wt/tg^ mice. First wave: 4.2 ± 0.2 *vs.* 4.5 ± 0.1 mm/min; second wave: 5.5 ± 1.4 *vs.* 3.1 ± 0.2 mm/min. (**F**) Comparison of CSD Ca^2+^ wave duration between IP_3_R2^+/−^;G7NG817^wt/tg^ and IP_3_R2^−/−^;G7NG817^wt/tg^ mice. First wave: 49.3 ± 5.4 *vs.* 50.9 ± 8.0 s; second wave: 35.0 ± 3.8 *vs.* 37.4 ± 11.6 s. (**G**) Comparison of inter-CSD Ca^2+^ wave interval between IP_3_R2^+/−^;G7NG817^wt/tg^ and IP_3_R2^−/−^;G7NG817^wt/tg^ mice. 353.5 ± 20.9 s *vs.* 386.6 ± 57.1 s, N = 6 *vs.* N = 4, p = 0.54. (**H**) Comparison of first and second CSD Ca^2+^ wave onset time between IP_3_R2^+/−^;G7NG817^wt/tg^ (WT, black) and IP_3_R2^−/−^;G7NG817^wt/tg^ (IP_3_R2 KO, blue) mice. First wave: 38.3 ± 2.6 *vs.* 45.0 ± 3.2 s, N = 10 *vs.* N = 8; second wave: 391.4 ± 22.1 *vs.* 434.3 ± 59.5 s, N = 7 *vs.* N = 4. (**I**) Comparison of baseline amplitude before AdR blocker in IP_3_R2^−/−^;G7NG817^wt/tg^ mice. (**J**) Effect of AdR blocker pretreatment on the recovery of neural oscillations after KCl-induced CSD in IP_3_R2^−/−^;G7NG817^wt/tg^ mice. Recovery is facilitated by AdR blocker pretreatment (N = 6) compared with the untreated control group (N = 6). (**K**,**L**) Comparisons of mean somatic and gliopil Ca^2+^ probability in IP_3_R2 KO expressing G-CaMP7 in astrocytes via AAV (**I**, 80 cells *vs.* 113 cells from N = 3 mice) and gliopil Ca^2+^ events in IP_3_R2 KO mice (**J**, N = 3 mice). *p < 0.05.

IP_3_-mediated astrocytic Ca^2+^ signaling accompanies the CSDs occurring in several neurologic conditions, although its possible role in neuroprotection remains controversial. We next used G7NG817 mice, that lack the astrocytic IP_3_ signaling pathway, to investigate the role of this pathway in the enhanced post-CSD recovery of neural activity with AdR blocker pretreatment. We bred IP_3_R2 KO / G7NG817 double transgenic mice and examined their neural activity by Ca^2+^ imaging before and after KCl-induced CSD (Fig. 3A–C). We found that CSD waves in IP_3_R2^−/−^;G7NG817^wt/tg^ mice were similar to those in IP_3_R2^+/−^;G7NG817^wt/tg^ mice in all aspects that we investigated including the number of CSD events, propagation speed, duration, inter-CSD interval, and onset (Fig. 3D–H). On the other hand, the recovery of neural activity was less pronounced in IP_3_R2^−/−^;G7NG817^wt/tg^ mice than in IP3R2^+/−^;G7NG817^wt/tg^ mice (Fig. 3B,J, 80.3 ± 2.3% *vs.* 66.5 ± 4.4%, p = 0.024). Intriguingly, AdR blocker pretreatment also boosted recovery of CSD waves in IP_3_R2^−/−^;G7NG817^wt/tg^ mice (Fig. 3C,J, 45 min after CSD, 76.6 ± 7.9% *vs.* 96.7 ± 4.1%, p = 0.017) without affecting the baseline amplitude (Fig. 3I, 2.7 ± 0.08% *vs.* 2.69 ± 0.08%).

It has been reported that gliopil Ca^2+^ events can occur independently of IP_3_R-mediated signaling^34,35^. To investigate whether post-CSD somatic and neurogliopil activities involve Ca^2+^ release from the astrocyte endoplasmic reticulum (ER), we expressed G-CaMP7 in astrocytes using AAV-GFAP-G-CaMP7 in IP_3_R2 KO mice, in which the major astrocytic ER IP_3_ receptor/Ca^2+^ channel IP_3_R2 is knocked out. Two-photon imaging revealed that somatic Ca^2+^ events were rarely observed in these mice, while aberrant gliopil Ca^2+^ activities were readily observable after CSD (Fig. 3K,L). Together with the G7NG817 findings, these results suggest that a mechanism independent of the astrocytic IP_3_/ Ca^2+^ pathway underlies the enhancement of AdR blocker-induced post-CSD recovery.

### AdR antagonism facilitates CSD recovery and K^+^ clearance

**Figure 4:**
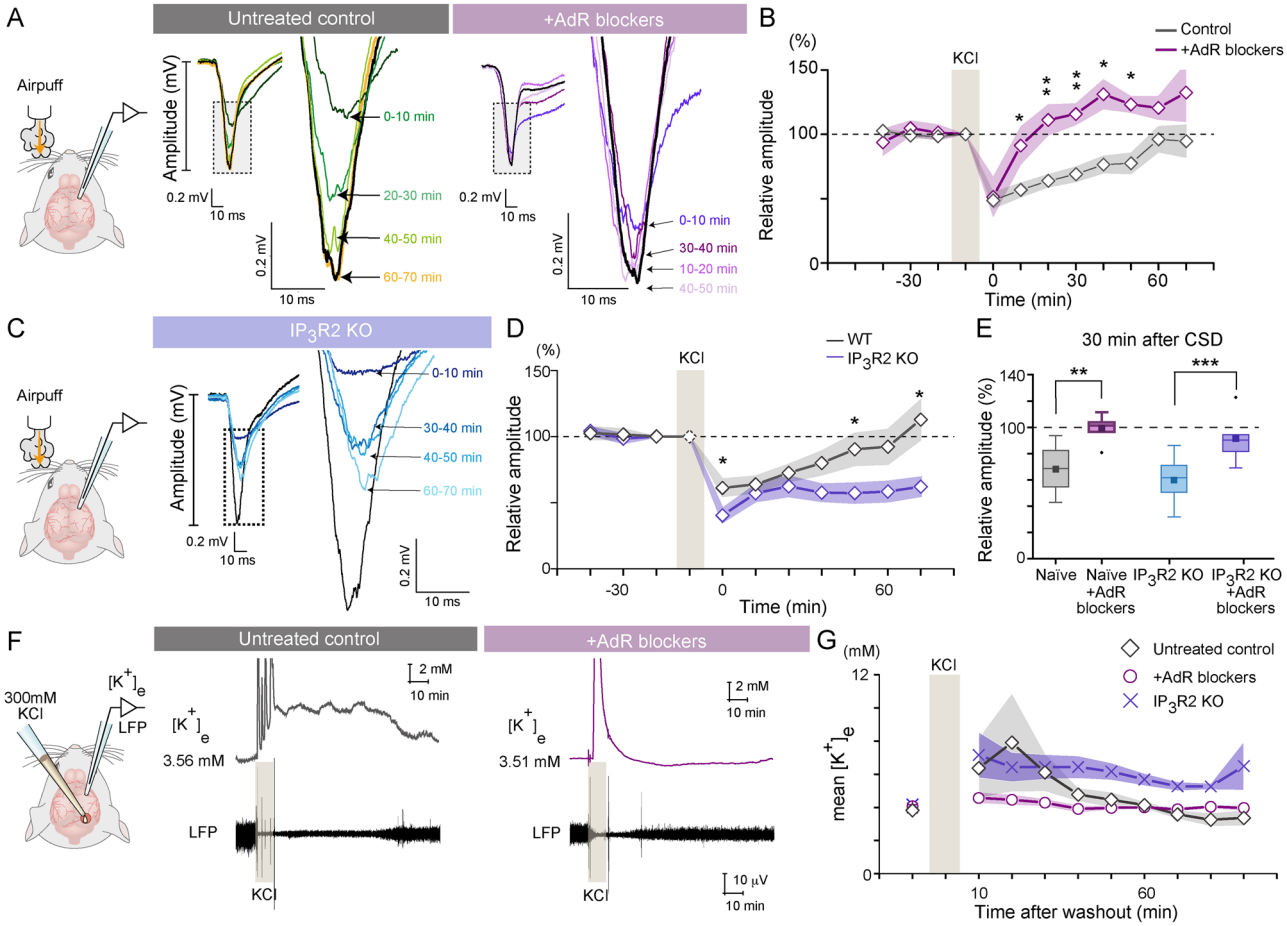
AdR antagonism enhances recovery of post-CSD neural activity and facilitates K^+^ clearance. (**A**) Mean traces of whisker-evoked barrel cortex layer 2/3 LFP recorded before (black) and after CSD (colors). Insets: full-range LFP waveforms. Dotted rectangular areas are magnified on the right. A mouse pretreated with AdR blockers shows similar LFP responses before and 30–50 min after CSD (black, orange and purple lines, respectively). (**B**) Effect of AdR blocker pretreatment on the recovery of whisker-evoked LFP after KCl-induced CSD. AdR blocker pretreatment (N = 6) vs. untreated control group (N = 7). (**C**) Mean whisker-evoked LFP traces measured in IP_3_R2 KO mice. (**D**) Comparison of the recovery of whisker response after KCl-induced CSD between WT mice (N = 7) and IP_3_R2 KO mice (N = 6). (**E**) Amplitudes of whisker-evoked LFP 30 min after KCl removal normalized to amplitude in the pre-KCl period. WT (N = 7), AdR blocker-pretreated WT (N = 6), IP_3_R2 KO (N = 6), and AdR blocker-pretreated IP_3_R2 KO mice (N = 7). (**F**) In vivo [K^+^]_e_ measurement and LFP recording were made simultaneously in the somatosensory cortex 2 mm away from the KCl application site. The example traces show that [K^+^]_e_ is elevated upon KCl application and gradually recovers after KCl removal. Spontaneous LFP activity is low while [K^+^]_e_ is high (left). AdR blocker pretreatment does not have profound effects on basal [K^+^]_e_ or LFP, but facilitates K^+^ clearance and LFP recovery after KCl removal (right). (**G**) Effect of AdR blocker pretreatment on K^+^ clearance. Median values for 10-min intervals are plotted. Untreated control (diamond, N = 8), and AdR blocker-treated mice (circle, N = 6), IP_3_R2 KO (cross, N = 6), Shades on line plots represent the area within mean ± SEM. *p < 0.05, **p < 0.01.

To investigate the functional response of neuronal activity, we evaluated the whisker stimulation-evoked LFP in the barrel cortex (~ 2 mm away from the CSD origin) of untreated controls, IP_3_R2KO mice, and mice pretreated with AdR blockers (Fig. 4A–E). As expected, whisker-evoked LFP amplitude was attenuated by ~ 50% after CSD and gradually recovered within 60 min in untreated mice (Fig. 4B, 61.3 ± 6.7% of baseline at 10 min vs. 92.2 ± 13.3% at 60 min). By contrast, mice pretreated with AdR blockers had complete restoration of the LFP amplitude in response to whisker stroking in only 30 min (untreated *vs.* pretreated C57BL/6 mice; 69.0 ± 6.0 *vs.* 108.6 ± 9.9%, two-way repeated-measures ANOVA, F(1,11) = 2.42, p = 0.0093, Tukey’s *post-hoc* analysis, p = 0.0024).

In other studies of the functional recovery of neural activity after CSD, we measured the amplitude of the LFP responses in the barrel cortex to whisker stimulation of IP_3_R2 KO mice. In these mice, the whisker-evoked potential amplitude was attenuated by ~ 50% after CSD and took a longer time to recover—more than 180 min (Fig. 4D, 61.3 ± 6.7% at 10 min vs. 92.2 ± 13.3% at 60 min). Again, pretreatment with AdR blockers significantly facilitated the recovery of LFP amplitude, which was almost fully restored within 30 min (Fig. 4E, 30 min after CSD, untreated WT vs. AdR blockers pretreated WT: 68.2 ± 4.6% vs. 99.2 ± 4.2%, p = 0.0014, untreated KO vs. AdR blockers pretreated KO: 59.8 ± 4.6% vs. 91.5 ± 6.2%, p = 0.0006).

Since the normalization of [K^+^]_e_ after CSD is a prerequisite for the recovery of neural activity, we hypothesized that AdR antagonism would promote the removal of excess K^+^ in the extracellular space. Accordingly, we recorded [K^+^]_e_ and LFP simultaneously in the primary somatosensory cortex (layer 2/3 of the barrel cortex, 2 mm away from the CSD origin) during and after CSD using double-barrel K^+^ electrodes (Fig. 4F). Plotting LFP amplitude *vs.* [K^+^]_e_ revealed an inverse relationship suggesting that post-CSD neural activity recovered in parallel with the restoration of extracellular ion concentrations^4^ (Suppl. Fig. S3). As shown in Fig. 4G, [K^+^]_e_ after CSD was consistently lower and the recovery of spontaneous LFP was faster in mice treated with AdR blockers than in control mice. Moreover, [K^+^]_e_ remained significantly higher even 1 h after KCl removal in IP_3_R2 KO mice (Fig. 4G, untreated control *vs.* IP_3_R2KO: 4.14 ± 0.22 mM *vs.* 5.68 ± 0.37 mM, p = 0.0011).

Collectively, these results provide supporting evidence that promotion of K^+^ clearance is the mechanism for the enhanced recovery from CSD by AdR pretreatment.

## Discussion

The G7NG817 transgenic mouse permits non-invasive monitoring of KCl-induced CSD episodes at macroscopic and microscopic levels. We here observed robust aberrant astrocytic Ca^2+^ activity after the passage of CSD waves in areas distal to the CSD initiation site, all in accordance with the prior report that abnormal AdR-dependent astrocytic Ca^2+^ activity also occurs in the setting of photothrombotic stroke^18^. By contrast, a recent ex vivo study examined post-CSD astrocytic Ca^2+^ activity close to cortical KCl application sites (~ 500 µm apart, 2.5 M KCl). That study found that antagonism of GABA-B receptors partially blocked astrocytic Ca^2+^ activity in the hippocampus^36^. While noradrenergic effects cannot be assessed in the acute slice preparations due to the ablation of the ascending adrenergic innervations, our in vivo experiments demonstrate a potent suppression by pretreatment with AdR blockers of the abnormal astrocytic Ca^2+^ activities in cortical regions otherwise occurring after passage of CSD waves (~ 2 mm from the site of KCl application).

Two-photon imaging in unanesthetized mice has shown that the majority of cortical astrocytic Ca^2+^ elevations are induced by alpha-1 adrenergic receptor activation^37^, prompting us to investigate the impact of adrenergic blockade after the passage of CSD waves. The asynchronous pattern of the Ca^2+^ activity in spatially separate astrocytes could be a reflection of fluctuations in the extracellular noradrenaline concentration or heterogeneous AdR sensitivity in individual astrocytes. Aberrant astrocytic Ca^2+^ activity has also been reported in various rodent brain disease models including ammonia neurotoxicity^33^, Alexander disease model mice^38^, epileptic seizures^39,40^, ischemic stroke^18,41^, and TBI^42^. Identification of the causes and impact of aberrant astrocytic Ca^2+^ activity is likely to contribute to therapeutic treatments for these conditions^43^. Of note, a recent optical assessment of extracellular noradrenaline levels indicated that high noradrenaline levels are sustained for minutes after activation of noradrenaline release in the cerebral cortex^44^. The relatively long persistence of elevated noradrenaline in the extracellular medium conceivably renders noradrenaline a more effective driver of the aberrant activity than certain other neurotransmitters that are rapidly taken up by transporters or enzymatically degraded. On this note, the current experiments have been performed under deep urethane anesthesia, whereby astrocytic activity and noradrenergic activity are largely suppressed^32^. It is tempting to speculate that the effects of AdR blockers on post-CSD recovery would be stronger in awake conditions.

Multiple factors have been identified to underlie CSD-induced functional depression including depolarization block by [K^+^]_e_ elevation^45^, adenosine-mediated synaptic suppression^46^, and inhibitory shift in post-synaptic excitation/inhibition balance^47^. During CSD-induced functional depression, action potentials and synaptic activity are first suppressed due to the depolarization block induced by the excessive rise in [K^+^]_e_ and the compromising of K^+^ uptake mechanisms^48^. Since KCl application does not per se induce neuronal degeneration^49^ and prolonged compromise of brain energy metabolism, normalization of [K^+^]_e_ is one of the critical components for restoration of neuronal activity. Interestingly, we find that spontaneous neural Ca^2+^ activity (Fig. 1J) displays slower recovery than [K^+^]_e_ normalization (Fig. 4G), whereas spontaneous or sensory evoked LFP activities recover in parallel with [K^+^]_e_. We speculate that although highly positively correlated, G-CaMP7 signals are more sensitive to parameters not measured by extracellular electrodes, such as pH. Alternatively, it is possible that Ca^2+^ signaling (e.g., via NMDA receptors or voltage-gated Ca^2+^ channels) needs a longer time to recover.

We described [K^+^]_e_ elevations lasting tens of minutes after removal of KCl in the somatosensory cortex, which is corroborated by a prior report describing that the rodent somatosensory cortex is more susceptible to CSD and [K^+^]_e_ elevations than other cortical areas^48^. It would be of interest in this context to image the spatial dynamics of [K^+^]_e_ and correlate with post-CSD neural activity by macroscopic imaging. Such experiments might be technically feasible in the near future, as several research groups are developing novel genetic and organic K^+^ sensors^50–52^.

Previous literature has suggested that K^+^ uptake in astrocytes is stimulated by IP_3_R2-mediated astrocytic Ca^2+^ elevations^53^ or by adrenergic receptor activation^54^. Indeed, our observations indicated that K^+^ clearance was delayed in IP_3_R2 KO mice (Fig. 4D,E). Multiple preclinical studies have reported that astrocytic Ca^2+^ elevation due to post-stroke cortical depolarization leads to worse stroke outcomes^55,56^ (but see Rakers et al.^57^). Moreover, neuroprotective roles of IP_3_R2 in TBI^42^, photothrombosis^58^ and middle cerebral artery occlusion (MCAO)^55^ have also been reported. Decreased post-CSD astrocytic Ca^2+^ signaling could thus be considered to delay recovery and exacerbate brain damage in the face of an ischemic event. Paradoxically, we demonstrated that AdR antagonism, which inhibits astrocytic Ca^2+^ elevations, facilitates the recovery of post-CSD neuronal activity and normalization of [K^+^]_e_. Furthermore, the boosting of K^+^ clearance by AdR antagonism was observed both in wild type and IP_3_R2 KO mice. These observations suggest that IP_3_R2-mediated Ca^2+^ elevation alone is not sufficient to explain the enhancement of K^+^ clearance by AdR blockers.

AdR antagonism has been shown to trigger an enlargement of the interstitial space^59,60^, which in turn augments influx of cerebrospinal fluid via glymphatic transport^60^. We recently showed that AdR antagonism facilitates [K^+^]_e_ normalization after photothrombosis stroke by a possible enhancement of cerebrospinal fluid inflow^18^. The observations reported here are consistent with accelerated recovery of [K^+^]_e_ after passage of CSD waves, and suggest that astrocytic Ca^2+^ elevations induced by Gq-coupled alpha-1 AdR played only a minor role in [K^+^]_e_ buffering. We cannot, however, exclude the possibility that present observations are a consequence of developmental adaptations in mice with constitutive IP_3_R2 deletion. Interestingly, recent studies have shown that treatment with the beta-AdR blocker propranolol reduces ischemic stroke damage in mice, suggesting a significant role of beta AdRs in the normalization of extracellular ion balance^18,61^. Further study is warranted to characterize the efficacy of individual AdR blockers as well as their synergy and optimal dosages. While the molecular mechanism that bridges AdR antagonism and boosting of [K^+^]_e_ clearance with concurrent recovery of electrical activity remains to be determined, the present observations are consistent with the concept that adrenergic signaling suppresses cerebrospinal fluid exchange and delays recovery after CSD.

## Materials and methods

All experimental protocols were approved by the RIKEN Institutional Animal Care and Use Committee. All animal experiments were performed according to the guidelines for animal experimentation of RIKEN that conforms with the Fundamental Guidelines for Proper Conduct of Animal Experiment and Related Activities in Academic Research Institutions (Ministry of Education, Culture, Sports, Science and Technology, Japan). Efforts were taken to minimize the number of animals used. This study was carried out in compliance with the ARRIVE guidelines.

### Animals

Adult male and female C57BL/6, G7NG817^26^, and IP_3_R2 KO^62^ mice were used (older than 8 weeks). The background strain of G7NG817 and IP_3_R2 KO mice is C57BL/6. Mice were housed under a 12 h /12 h light/dark cycle and raised in groups of up to five mice. G7NG817 and IP_3_R2 KO mice are available from the RIKEN BioResource Center (Resource IDs: RBRC09650 and RBRC10289, respectively).

### Surgical procedures

The surgical procedures described below are adapted from earlier works of the laboratory^18,26,63,64^. Mice were anesthetized with urethane (1.6 g/kg) and body temperature was maintained at 37 °C with a heating pad (BWT-100A, BioResearch Center or TR-200, Fine Science Tools) during surgery and recording. For transcranial imaging, the skull was treated with a mixture of paraffin oil and Vaseline (1:1) immediately after the excision of the scalp, to increase its transparency.

For two-photon imaging, a metal frame was attached to the skull using dental acrylic cement (Fuji LUTE BC, GC Corporation, Super Bond C&B, Sun Medical). A craniotomy (2 mm in diameter) was made above the somatosensory cortex (AP − 1.0 mm, ML 1.0 mm). The dura mater was surgically removed. Sulforhodamine 101 (100 µM in PBS) was topically applied to label astrocytes and the cortical surface was washed with HEPES-buffered artificial cerebrospinal fluid (HEPES-ACSF) after 1 min. After the dye loading, the craniotomy was covered with agarose (1.5% w/v in ACSF) and gently sealed with a thin glass coverslip (3 mm × 3 mm, thickness: 0.12 mm, Matsunami Glass.) The cranial window was secured with dental cement.

For experiments involving local field potential recording, a screw electrode (diameter, 0.7 mm; SUS-XM7, no. 00PH+14046, Matsumoto Industry) was implanted in the interparietal bone to serve as a reference electrode.

### AdR blockers

The combination of AdR antagonists used throughout the current study consisted of propranolol (10 mg/kg), prazosin (10 mg/kg), and atipamezole (1 mg/kg). These antagonists were prepared as a 0.1% solution of each drug in saline and administered separately by i.p. injection 30 min before CSD induction.

### In vivo transcranial fluorescence imaging

In vivo transcranial fluorescence imaging was performed as described earlier in Monai et al.^26^. Urethane-anesthetized mice were fixed to a stereotaxic stage by securing the ear canals and incisors, and then placed under a fluorescence stereomicroscope (MZ10F, Leica). A GFP3 filter set (excitation 470 ± 20 nm, emission 525 ± 25 nm, Leica) was used with an EL6000 light source (Leica). Images were acquired using an ORCA-Flash 4.0 CMOS camera (Hamamatsu Photonics) and HC Image software (Hamamatsu Photonics, image size: 512 × 512 pixels, pixel depth: 16 bit, frame rate: 10 Hz).

### In vivo two-photon imaging

Two-photon imaging was performed on urethane-anesthetized adult mice (as above) using a resonant scanner-based B-Scope (Thorlabs) with a Chameleon Vision 2 laser (coherent, wavelength 920 nm) and an Olympus objective lens (XLPlan N 25×) as described before^18,26^. The B-Scope is equipped with a reverse dichroic mirror (ZT405/488/561/680-1100rpc, Chroma) and the emission light was separated by a dichroic mirror (FF562-Di03, Semrock) with bandpass filters FF03-525/50 and FF01-607/70 (both from Semrock) for the green and red channels, respectively. Images were acquired using the ThorImage software at a frame rate of 30 Hz.

### Sensory stimulation

For whisker-evoked response experiments, single air-puffs (70 kPa, 10 ms) were applied to the left whisker pad at intervals of 30 s (Fig. 4A–E).

### Local field potential recording

Extracellular recordings were performed with an ELC-03XS amplifier (NPI electronic). A glass micropipette (10 µm tip diameter, 1B150F-4, World Precision Instruments) was filled with HEPES-ACSF (pH 7.4) and placed in an electrode holder with a head-stage preamplifier. The head-stage was then mounted to a remote-controlled micromanipulator (Sensapix). Under a stereomicroscope, the glass micropipette was inserted into the primary visual cortex (250 µm below the pia) at a 30-degree insertion angle. For stabilization of evoked responses, recording sessions started 1–2 h after insertion of the electrode^63,64^. After amplification (2000×, 0.1 Hz–3 kHz), the signal was digitized at 20 kHz and stored on a hard drive using a LabVIEW-based data acquisition system. The field potential experiments were performed under room light conditions.

For simultaneous LFP recording and cortical imaging, urethane-anesthetized mice were fixed to a stereotaxic stage, and a 16-channel linear silicon probe (inter-channel distance = 50 µm; Alx15-5 mim-50-177-A16; NeuroNexus, Ann Arbor, MI, USA) was inserted in somatosensory cortex of the right hemisphere (Bregma: mediolateral 4.0 mm, anteroposterior − 1.0 mm). The electrode was tilted at a 60° angle toward the anterior direction, and the tip of the electrode was inserted at the depth of 900 µm from the surface of the cortex. Extracellular field potentials were recorded continuously at 24.4 kHz with an RZ2 multi-channel recording system (Tucker-Davis Technologies, Alachua, FL, USA). Electrophysiological recording was synchronized with imaging by feeding 25 Hz image acquisition TTL pulses. Body temperature was maintained at 37C throughout the surgery and recording sessions by a heat pad with rectal temperature feedback. Transcranial G-CaMP7 fluorescent intensities were measured from the location of electrode and the corresponding area of the same side of the cortex.

### Extracellular potassium recording

Extracellular recordings were performed as described earlier in Monai et al.^18^. Ion-sensitive microelectrodes for measuring extracellular K^+^ were made from double-barreled glass pipettes (A-M SYSTEMS, INC., 607000) with a tip diameter of < 10 µm using a pipette puller (P-97, Sutter). Pipettes were silanized by vaporizing dimethylsilane in a small container for 1 h at 200 °C. One or the other tip of the electrodes was loaded with 2.5 µl of valinomycin-based K^+^ ion-exchange resin (potassium ionophore I—cocktail B, front-loaded) and subsequently filled with 150 mM KCl. The other tip was filled with 150 mM NaCl and used as a reference for the LFP recording.

K^+^-sensitive electrodes were calibrated before and after each experiment using a set of solutions with known K^+^ concentration (3.5, 4.5, 10, 20, 50, and 100 mM). Each electrode was calibrated by calculating the least squares linear regression slope between the measured voltage and the known K^+^ concentration.

In vivo extracellular K^+^ recordings from cortical layer 2/3 (250 µm from the surface) were made using a DC amplifier (MultiClamp 700B, Axon Instruments) and recorded at a 20 kHz sampling rate. Simultaneous recordings were made from K^+^-sensitive and reference LFP electrodes. The reference LFP was subtracted from the K^+^-sensitive electrode recordings, and the resulting signal was resampled to 10 Hz and converted to K^+^ concentration (mM) according to the calibration plot.

### Data analysis

#### Transcranial imaging

The original 512 × 512 pixel images were reduced to 64 × 64 pixels by binning. The baseline F is defined as the mean intensity of the 40-s period ending 20 s prior to KCl application. CSD onset is defined when the signal amplitude exceeded + 5 SD above the baseline (Fig. 3D). The peak value was detected by spline interpolation. The signal amplitude was calculated as the root mean square of signal intensity for each 5-min window after applying a bandpass filter (0.1–3 Hz) (Figs. 1H, 3G).

CSD speed was calculated as the time taken for the CSD to propagate from a ROI close to the KCl application site to an ROI positioned 2 mm anteriorly. The arrival time of CSD wave was determined as the time point when the signal reached the half-maximum of the peak amplitude of the ROI (Fig. 1E–G). The passage of CSD wave was determined as the time point when amplitude had subsided to the half-maximum of the ROI peak amplitude (Fig. 1H).

#### Two-photon imaging

For each pixel, relative fluorescence changes (ΔF/F) were computed as follows: F, the baseline mean, was defined as the mean intensity of the pre-CSD period; ΔF was the difference between the signal and F. ROIs for astrocyte somata were selected manually within the SR101-positive area. ROIs for neurogliopil or gliopil were selected as the areas showing Ca^2+^ events at least once during before CSD periods (92 ± 13 s, e.g., Fig. 2A, panel 1) or during the interval between the first and the second CSD waves (264 ± 34 s between the first and second CSD episodes, e.g., Fig. 2A, panel 3–5). Pixels that had intensities larger than the mean + 4SD were considered to be high-Ca^2+^ event. Ca^2+^ event probability (P(High Ca^2+^)) was calculated for soma and non-soma ROIs separately. For somata, P(High Ca^2+^) was computed for each soma by dividing the total high-Ca^2+^ pixel count by the total number of the soma pixels and time (Figs. 2D, 3K). For neurogliopil and gliopil, P(High Ca^2+^) was computed for the entire non-soma ROI (Figs. 2E, 3J).

### Statistics

Mean values are presented with the standard error of the mean (SEM). Shaded areas on line plots represent the areas within mean ± SEM. For comparisons of two sample means, two-sample t tests were used. Multiple group comparisons were performed by one-way or two-way analysis of variance (ANOVA) followed by Tukey’s *post-hoc* analysis. All statistical tests were computed using ORIGIN (OriginLab) or R (ANOVA-kun).

## Supplementary Information

Supplementary Video 1.

Supplementary Video 2.

Supplementary Information 1.

## Data Availability

All transgenic mice used in this manuscript are available from RIKEN BioResource Center.
