# Author Correction: Adrenergic inhibition facilitates normalization of extracellular potassium after cortical spreading depolarization

**DOI:** 10.1038/s41598-022-08615-0

**Published:** 2022-03-18

**Authors:** Hiromu Monai, Shinnosuke Koketsu, Yoshiaki Shinohara, Takatoshi Ueki, Peter Kusk, Natalie L. Hauglund, Andrew J. Samson, Maiken Nedergaard, Hajime Hirase

**Affiliations:** 1 Laboratory for Neuron-Glia Circuitry, RIKEN Center for Brain Science, Wako, Saitama 351-0198 Japan; 2 Faculty of Core Research Natural Science Division, Ochanomizu University, Bunkyo-Ku, Tokyo, 112-8610 Japan; 3 Department of Integrative Anatomy, Nagoya City University Graduate School of Medical Sciences, Nagoya, Aichi 467-8601 Japan; 4 Division of Histology and Cell Biology, Department of Anatomy, Jichi Medical University, Shimotsuke, Tochigi 329-0498 Japan; 5 Center for Translational Neuromedicine, Faculty of Health and Medical Sciences, University of Copenhagen, 2200 Copenhagen, Denmark; 6 Center for Translational Neuromedicine, University of Rochester Medical Center, Elmwood Avenue 601, Rochester, NY 14642 USA

Correction to: *Scientific Reports* 10.1038/s41598-021-87609-w, published online 14 April 2021

The original version of this Article contained errors in Figure 3 (**B**) and (**C**), where grey shadings were incorrectly positioned.

The original Figure 3 and accompanying legend appear below.

**Figure 3:**
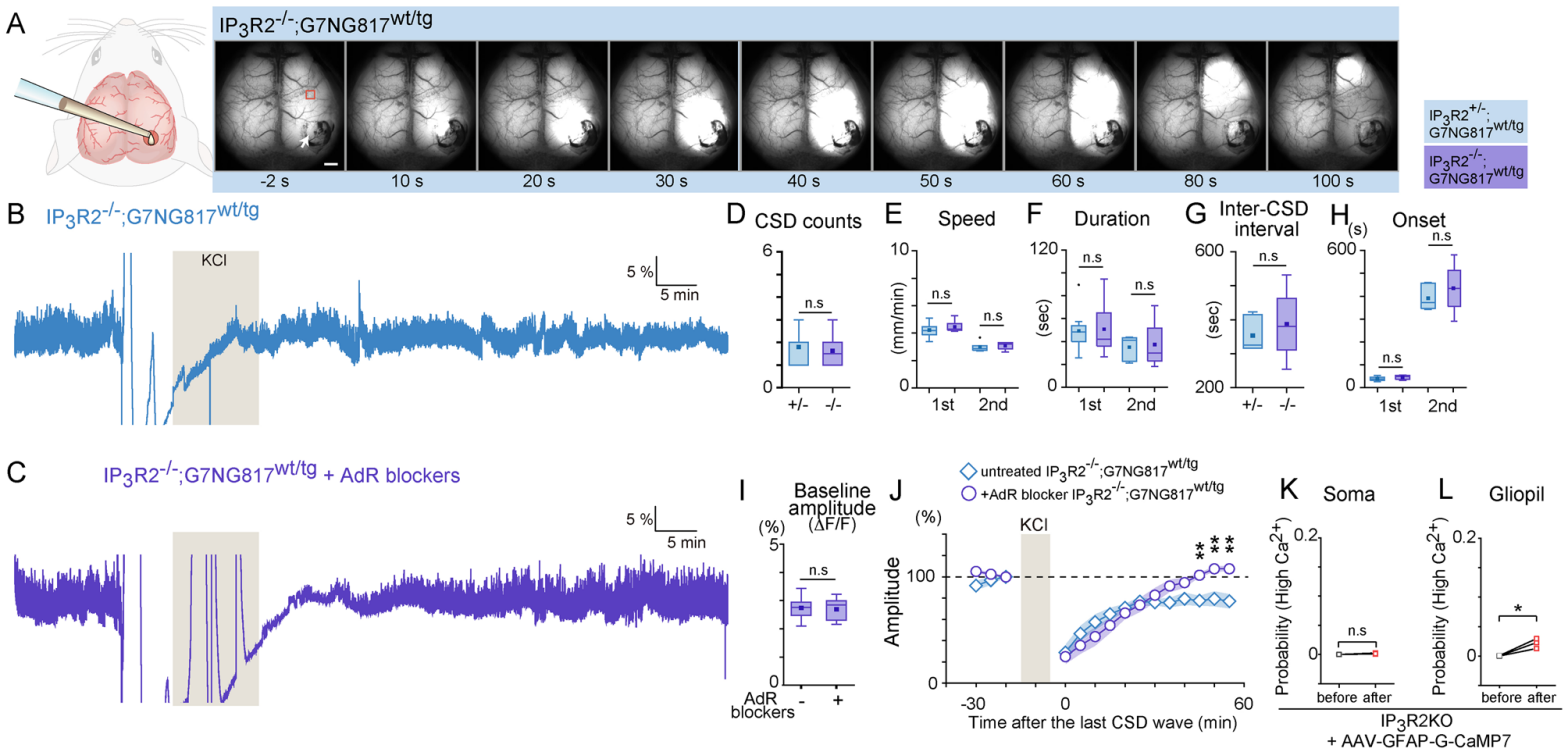
CSD propagation and neural activity recovery in IP_3_R2 KO mice. (**A**) Representative image of the time series of CSD propagation. Other than using IP_3_R2^−/−^;G7NG817^wt/tg^ double transgenic mouse as subjects, the experimental conditions are the same as in Fig. 1. Scale bar 1 mm. (**B**) Example trace of Ca^2+^ activity of an ROI located ~ 2 mm anterior to the KCl application site (Black square indicated in **A**). Note that neural activity does not recover completely within 50 min. (**C**) Similar Ca^2+^ signal trace as (**B**). measured in an IP_3_R2^−/−^;G7NG817^wt/tg^ mouse pretreated with AdR blockers. (**D**) Comparison of CSD Ca^2+^ wave number during 10 min KCl application between IP_3_R2^+/−^;G7NG817^wt/tg^ and IP_3_R2^−/−^:G7NG817^wt/tg^ mice. 1.8 ± 0.2 *vs.* 1.6 ± 0.3, from N = 10 *vs.* N = 8, p = 0.64. (**E**) Comparison of CSD Ca^2+^ propagation speed between IP_3_R2^+/−^;G7NG817^wt/tg^ and IP_3_R2^−/−^:G7NG817^wt/tg^ mice. First wave: 4.2 ± 0.2 *vs.* 4.5 ± 0.1 mm/min; second wave: 5.5 ± 1.4 *vs.* 3.1 ± 0.2 mm/min. (**F**) Comparison of CSD Ca^2+^ wave duration between IP_3_R2^+/−^;G7NG817^wt/tg^ and IP_3_R2^−/−^;G7NG817^wt/tg^ mice. First wave: 49.3 ± 5.4 *vs.* 50.9 ± 8.0 s; second wave: 35.0 ± 3.8 *vs.* 37.4 ± 11.6 s. (**G**) Comparison of inter-CSD Ca^2+^ wave interval between IP_3_R2^+/−^;G7NG817^wt/tg^ and IP_3_R2^−/−^;G7NG817^wt/tg^ mice. 353.5 ± 20.9 s *vs.* 386.6 ± 57.1 s, N = 6 *vs.* N = 4, p = 0.54. (**H**) Comparison of first and second CSD Ca^2+^ wave onset time between IP_3_R2^+/−^;G7NG817^wt/tg^ (WT, black) and IP_3_R2^−/−^;G7NG817^wt/tg^ (IP_3_R2 KO, blue) mice. First wave: 38.3 ± 2.6 *vs.* 45.0 ± 3.2 s, N = 10 *vs.* N = 8; second wave: 391.4 ± 22.1 *vs.* 434.3 ± 59.5 s, N = 7 *vs.* N = 4. (**I**) Comparison of baseline amplitude before AdR blocker in IP_3_R2^−/−^;G7NG817^wt/tg^ mice. (**J**) Effect of AdR blocker pretreatment on the recovery of neural oscillations after KCl-induced CSD in IP_3_R2^−/−^;G7NG817^wt/tg^ mice. Recovery is facilitated by AdR blocker pretreatment (N = 6) compared with the untreated control group (N = 6). (**K**,**L**) Comparisons of mean somatic and gliopil Ca^2+^ probability in IP_3_R2 KO expressing G-CaMP7 in astrocytes via AAV (**I**, 80 cells *vs.* 113 cells from N = 3 mice) and gliopil Ca^2+^ events in IP_3_R2 KO mice (**J**, N = 3 mice). *p < 0.05.

The original Article has been corrected.

