# Supplementary material for: Adrenergic inhibition facilitates normalization of extracellular potassium after cortical spreading depolarization
Source: Sci Rep. 2021 Apr 14;11:8150. doi: 10.1038/s41598-021-87609-w (PMC8047013; doi:10.1038/s41598-021-87609-w)
Supplement: Supplementary file 3 — Supplementary Information 1. [file 41598_2021_87609_MOESM3_ESM.pdf]

Supplementary Information

Adrenergic inhibition facilitates normalization of extracellular potassium after cortical spreading depolarization

Hiromu Monai[1,2]*, Shinnosuke Koketsu[3], Yoshiaki Shinohara[3,4], Takatoshi Ueki[3], Peter Kusk[5], Natalie L Hauglund[5], Andrew J Samson[5], Maiken Nedergaard[5,6], Hajime Hirase[1,5]*

Affiliations:

1. Laboratory for Neuron–Glia Circuitry, RIKEN Center for Brain Science, Wako, Saitama, 351-0198, Japan
2. Faculty of Core Research Natural Science Division, Ochanomizu University, Bunkyo-Ku, Tokyo, 112-8610, Japan
3. Department of Integrative Anatomy, Nagoya City University Graduate School of Medical Sciences, Nagoya, Aichi, 467-8601, Japan
4. Division of Histology and Cell Biology, Department of Anatomy, Jichi Medical University, Shimotsuke, Tochigi, 329-0498, Japan
5. Center for Translational Neuromedicine, Faculty of Health and Medical Sciences, University of Copenhagen, 2200 Copenhagen, Denmark
6. Center for Translational Neuromedicine, University of Rochester Medical Center, Elmwood Avenue 601, Rochester, NY 14642, USA;

Supplementary figures

Supplementary Figure S1

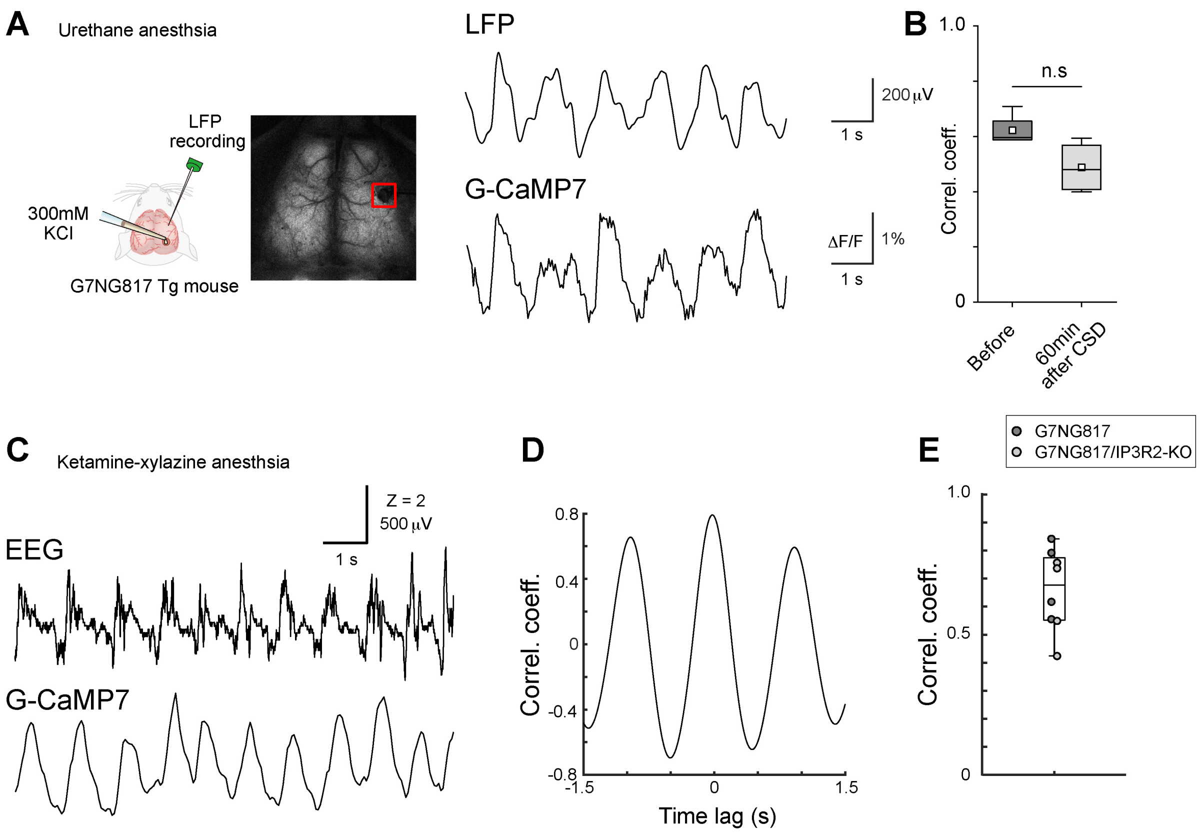

Supplementary Figure S1. Transcranial G-CaMP7 signal reflects cortical LFP and EEG in G7NG817 mice.

A. Cortical local filed potential (LFP; low-pass filtered at 2 Hz) and G-CaMP7 fluorescence were simultaneously measured from the right somatosensory cortex (red square) during deep urethane anesthesia. Representative LFP and G-CaMP7 traces showing spontaneous up/down states are plotted..

B. Comparison of the correlation coefficient between LFPs and Ca^{2+} signal before and 60 min after KCl-induced CSD (0.62 ± 0.029 vs. 0.49 ± 0.048, N = 4 mice each, p = 0.14).

C. Representative cortical surface EEG and G-CaMP7 fluorescence simultaneously monitored during ketamine-xylazine anesthesia.

D. Cross-correlogram between EEG and G-CaMP7 signals recorded from the mouse presented in C.

E. Summary of the peak correlation coefficients of EEG and G-CaMP7 signals from G7NG817 mice and $IP_3R2^{-/-}$;G7NG817 mice (0.70 ± 0.069 vs. 0.62 ± 0.075, N=4 mice each, p = 0.45).

Supplementary Figure S2

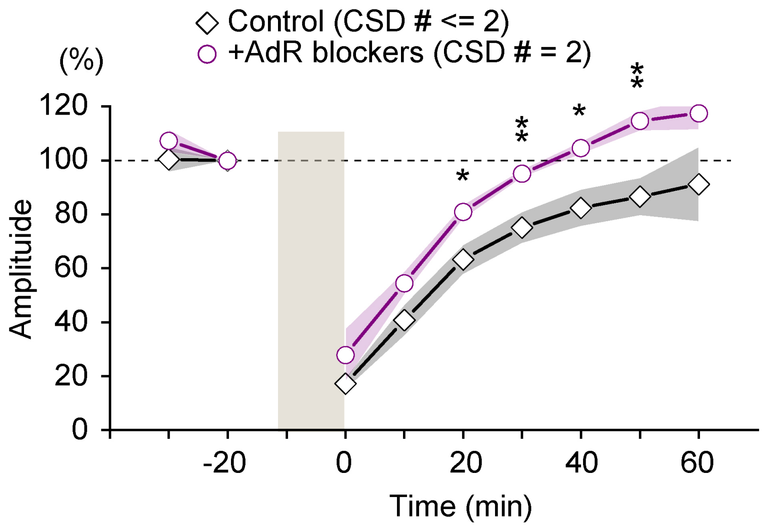

Supplementary Figure S2. AdR blocker pretreatment accelerates post-CSD neural activity recovery compared with non-treated control mice that had equal or fewer CSD waves. Post-CSD neural activity recovery is plotted for the AdR blocker pretreated group that had two CSD events (N = 3, circle) and the non-treated control group that had one or two CSDs (N= 3, diamond).

*p<0.05, **p<0.01, ***p<0.001, Error bars are SEM.

Supplementary Figure S3

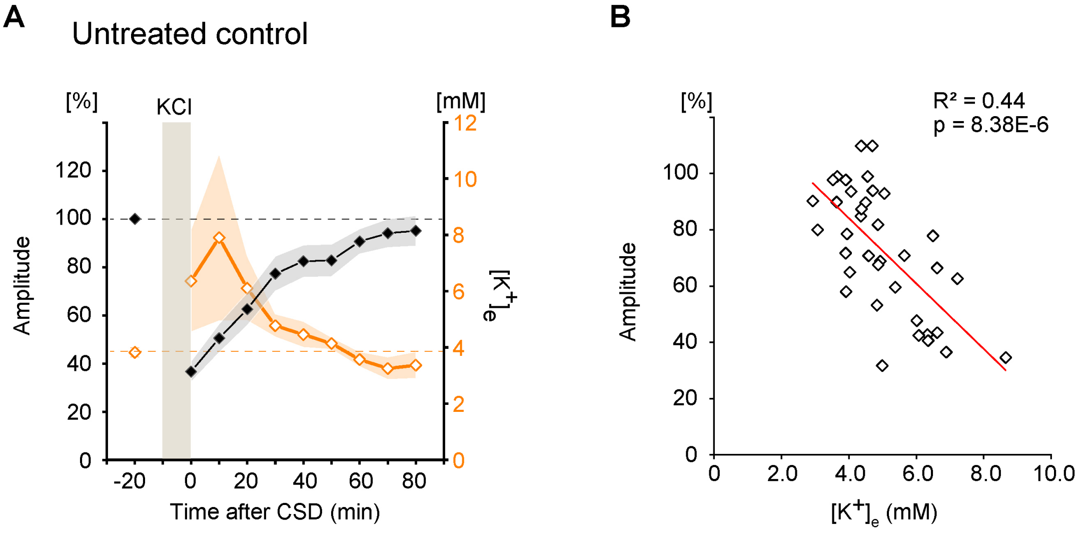

Supplementary Figure S3. Spontaneous LFP amplitude and $[K^+]_e$ are negatively correlated in post-CSD neural activity recovery periods.

A. Simultaneous *in vivo* $[K^+]_e$ (blank diamond) and spontaneous LFP recording (filled diamond) in untreated control group (N =8). Median values for 10-min intervals are plotted. Shades on line plots represent the area within mean ± SEM.

B. Correlation between spontaneous LFP amplitude and $[K^+]_e$ for periods 10 min after KCl removal (untreated control, diamond, N = 8)

Supplementary video legends

Supplementary Video S1

Transcranial imaging of KCl-induced CSD Ca^{2+} waves in an anesthetized G7NG817 mouse (As presented in Fig. 1A-B). KCl concentration: 300 mM, urethane dosage: 1.7 g/kg, body temperature: 37.0 °C. The video plays 30 times faster than real time.

Supplementary Video S2

Neuronal and astrocytic Ca^{2+} activity in layer 2/3 of somatosensory cortex in an anesthetized G7NG817 mouse. Each panel corresponds to a different time point in a KCl-induced CSD experiment (i.e. pre-CSD, CSD, and post-CSD). Image contrast is adjusted during CSD (center) to cover high-intensity pixels. Urethane dosage: 1.7 g/kg, body temperature: 37.0 °C. The video plays 15 times faster than real time.
